# Anchoring Gated Mesoporous Silica Particles to Ethylene Vinyl Alcohol Films for Smart Packaging Applications

**DOI:** 10.3390/nano8100865

**Published:** 2018-10-22

**Authors:** Virginia Muriel-Galet, Édgar Pérez-Esteve, María Ruiz-Rico, Ramón Martínez-Máñez, José Manuel Barat, Pilar Hernández-Muñoz, Rafael Gavara

**Affiliations:** 1 Instituto de Agroquímica y Tecnología de Alimentos, IATA-CSIC, Grupo de Envases, Av. Agustin Escardino 7, 46980 Paterna, Spain; vmurielgalet@gmail.com (V.M.-G.); phernan@iata.csic.es (P.H.-M.); 2 Departamento de Tecnología de Alimentos, Grupo de Investigación e Innovación Alimentaria, Universitat Politècnica de València. Camino de Vera s/n, 46022 Valencia, Spain; edpees@upv.es (É.P.-E.); maruiri@etsia.upv.es (M.R.-R.); jmbarat@tal.upv.es (J.M.B.); 3 Instituto Interuniversitario de Investigación de Reconocimiento Molecular y Desarrollo Tecnológico (IDM), Universitat Politècnica de València, Universitat de València. Departamento de Química, Universitat Politècnica de València, Camino de Vera s/n, 46022 Valencia, Spain; rmaez@qim.upv.es; 4 CIBER de Bioingeniería, Biomateriales y Nanomedicina (CIBER-BBN), Camino de Vera s/n, 46022 Valencia, Spain

**Keywords:** MCM-41, gated mesoporous silica particles, EVOH films, anchorage on film surface, active packaging, pH-mediated delivery

## Abstract

This work is a proof of concept for the design of active packaging materials based on the anchorage of gated mesoporous silica particles with a pH triggering mechanism to a packaging film surface. Mesoporous silica micro- and nanoparticles were loaded with rhodamine B and functionalized with *N*-(3-trimethoxysilylpropyl)diethylenetriamine. This simple system allows regulation of cargo delivery as a function of the pH of the environment. In parallel, poly(ethylene-*co*-vinyl alcohol) films, EVOH 32 and EVOH 44, were ultraviolet (UV) irradiated to convert hydroxyl moieties of the polymer chains into –COOH functional groups. The highest COOH surface concentration was obtained for EVOH 32 after 15 min of UV irradiation. Anchoring of the gated mesoporous particles to the films was carried out successfully at pH 3 and pH 5. Mesoporous particles were distributed homogeneously throughout the film surface and in greater concentration for the EVOH 32 films. Films with the anchored particles were exposed to two liquid media simulating acidic food and neutral food. The films released the cargo at neutral pH but kept the dye locked at acidic pH. The best results were obtained for EVOH 32 irradiated for 15 min, treated for particle attachment at pH 3, and with mesoporous silica nanoparticles. This opens the possibility of designing active materials loaded with antimicrobials, antioxidants, or aromatic compounds, which are released when the pH of the product approaches neutrality, as occurs, for instance, with the release of biogenic amines from fresh food products.

## 1. Introduction

Active packaging is a novel technology in which the packaging system is designed to actively improve the stability and/or quality of the packaged product from processing to consumption. This technology is being implemented in various industries, although, owing to the fast perishability of food, pharmaceutical, and cosmetic products, these are the areas that receive the most attention [1,2]. The mechanism of action is basically related to the release or retention of substances whose presence or absence is important for the product’s stability or quality. By suitably combining design and mechanism of action, various active packaging technologies were created, including oxygen scavengers, ethylene scavengers, humidity controllers, aroma releasers, antioxidant or antimicrobial releasers, enzyme-based systems, etc. Several interesting reviews were published on this subject [3,4].

There are basically two procedures for designing active packaging systems: manufacture of an independent device that contains the active agent [5], or manufacture of active materials that incorporate the agent in the package wall or on the wall surface [6]. Of these two general procedures, the manufacture of active packaging materials is gaining attention over the development of independent devices. In the latter, the presence of a device often labeled as toxic because it contains an inedible component in contact with the product is not well accepted by consumers. Thus, the incorporation of active agents on or in the polymeric films that constitute the package walls is the preferred option. Owing to their at least partially amorphous morphology and the presence of free volume (voids) in polymer matrices, polymeric films allow mass transport (permeation, migration, scalping, or sorption), processes that are profitable for the design of active materials [6]. However, some precautions have to be considered in these designs. Firstly, the incorporation of the active substance in the package walls or the action of the substance should not modify the functional properties of the packaging throughout its use. Secondly, the substance should not lose activity owing to interactions or degradations caused by the film manufacturing procedure. Thirdly, the mechanism of action (release, adsorption) should be maintained and controlled. Finally, and most importantly, the packaging system should include a triggering mechanism to avoid premature action and partial exhaustion of the system prior to the presence of the product to be protected. This last condition was achieved through various procedures: humidity-activated systems [7,8], temperature-activated systems [9], or radiation-activated systems [10]. Another approach focuses on including substances covalently anchored to the package walls that exert their action via direct contact with the packaged product, that is, no agent is released or captured. Such systems were successfully prepared, for instance, via oxidation of a conventional film surface and anchorage of enzymes [11,12], antimicrobials [13] (such as lysozyme), or antioxidants [14]. Indeed, Goddard, Talbert and Hotchkiss [11] successfully functionalized a polyethylene surface with lactase to design an active package that reduced the amount of lactose in milk. Muriel Galet, Talbert, Hernandez Munoz, Gavara and Goddard [12] anchored lysozymes to the surface of ethylene vinyl alcohol (EVOH) to generate a film with antimicrobial properties against *Listeria monocytogenes*. Similarly, Saini, Sillard, Belgacem and Bras [13] anchored a bacteriocin to cellulose fibers with potential application in active food packaging. Roman, Decker and Goddard [14] prepared an antioxidant active film via functionalization of a polypropylene surface with polyphenols generated by the action of laccase. Vasile, et al. [15] covalently bonded chitosan to plasma-treated polyethylene and obtained a material with antimicrobial properties against *Salmonella enteriditis*, *Escherichia coli*, and *Listeria monocytogenes.*

Considered from another point of view, new technologies based on nanomaterials or nanocomposites received massive attention in the packaging field lately, especially in active packaging research. The new properties and functions of nanoscale particles display new opportunities for enhancing traditional product performance. A wide range of nanostructured materials were included as fillers in packaging films to provide barrier properties or improved mechanical resistance, or to control activity in smart packaging applications [16,17]. Moreover, nanostructures were also included to provide antioxidant and antimicrobial properties. Biddeci, et al. [18] reported the design of a pectin-based biopolymer film with both antioxidant and antimicrobial activities. The film was created by filling the pectin matrix with modified halloysite nanotubes containing essential peppermint oil. Later, Li, et al. [19] used the solvent volatilization method to prepare polylactide films containing nanoparticles of silver and titanium dioxide. The developed films showed good antimicrobial activity against two common food pathogens: *E. coli* and *Listeria monocytogenes*.

Among commented nanostructured materials, mesoporous silica particles (MSPs) exhibit unique features such as high stability, biocompatibility, nontoxicity, and large load capacity. Moreover, the possibility of functionalizing the external surface with gate-like ensembles makes these materials unique candidates for the design of on-command controlled release devices. In fact, a number of gated materials based on mesoporous silica particles able to deliver the cargo upon application of target physical (such as light or temperature) [20,21], chemical (pH changes or redox potential) [22,23], and biochemical (enzymes, antibodies, or DNA) [24] stimuli were reported. These functionalized MSPs are mainly used in the fields of drug delivery [25,26,27,28] or sensing [29], and are prepared in the form of nanoparticles [30] or microparticles [31]. In contrast, gated MSPs are barely incorporated in polymers or on surfaces. Moreover, as far as we are aware, MSPs were never previously used in the design of active packaging.

In this scenario, we report herein the preparation of smart films based on poly(ethylene-*co*-vinyl alcohol) (EVOH)-containing gated MSPs covalently anchored and able to modulate the release of a model molecule in response to changes in the pH of the environment. EVOHs are a family of copolymers with different ethylene molar percentages, commonly used in packaging technologies as they provide an excellent oxygen barrier thanks to their high crystallinity ratio and the high cohesive energy density caused by the large number of hydrogen bonds between macromolecular chains. Moreover, EVOH not only provides hydrophilicity, but also contains suitable hydroxyl sites for functionalization [12].

In most reported works about the inclusion of micro- or nanoparticles in packaging systems, functional nanofillers are mixed with the polymer via three procedures: the solvent casting method, the melt mixing method, and in situ polymerization [32]. However, these procedures are not suitable for the present design because the MSP particles require to be exposed to conditions that would open the gates, promoting the release of the agent included in the particle during preparation. Moreover, if the particle was included in the polymer matrix, gate opening would be impeded or delayed.

## 2. Materials and Methods

### 2.1. Chemicals and Reagents

Tetraethylorthosilicate (TEOS), *N*-cetyltrimethylammonium bromide (CTABr), triethanolamine (TEAH3), sodium hydroxide (NaOH), acetonitrile, and *N*-(3-trimethoxysilylpropyl)diethylenetriamine (N3) were provided by Sigma (Sigma-Aldrich Química S.L., Madrid, Spain). Films, 75-μm-thick, of Soarnol DC3203FB ethylene vinyl alcohol copolymer with 32% ethylene molar content (EVOH 32) and Soarnol AT4403B with 44% ethylene molar content (EVOH 44) were kindly provided by The Nippon Synthetic Chemical Company (Osaka, Japan). Isopropanol, acetone, acetic acid, 1-ethyl-3-(3-dimethylaminopropyl) carbodiimide (EDC), *N*-hydroxysuccinimide (NHS), and Toluidine Blue O (TBO) were purchased from Sigma (Sigma-Aldrich, Madrid, Spain). Water was obtained from a Milli-Q Plus purification system (Millipore, Molsheim, France).

### 2.2. Synthesis of Mesoporous Silica Particless

Microparticulated MCM-41 particles (**M**) were synthesized following the so-called “atrane route”, according to the method described by Perez-Esteve, et al. [33]. *N*-cetyltrimethylammonium bromide (acting as a structure-directing agent) was added to a solution of triethanolamine (TEAH3) containing sodium hydroxide (NaOH) and tetraethylorthosilicate (TEOS). Temperature was then set at 118 °C. After the CTABr was dissolved in the solution, water was slowly added with vigorous stirring at 70 °C. This mixture was aged in an autoclave at 100 °C for 24 h. The molar ratio of the reagents was fixed at 7 TEAH3:2 TEOS:0.52 CTABr:0.5 NaOH:180 H_2_O.

Nanoparticulated MCM-41 particles (**N**) were synthesized using the procedure described by Perez-Esteve, Fuentes, Coll, Acosta, Bernardos, Amoros, Marcos, Sancenon, Martinez-Manez and Barat [33]. *N*-cetyltrimethylammonium bromide was firstly dissolved in 480 mL of deionized water. Then, 3.5 mL of a sodium hydroxide solution was then added, and the mixture was heated to 80 °C. Finally, TEOS was added dropwise to the surfactant solution. The mixture was stirred for 2 h to give a white precipitate. The molar ratio of the reagents was fixed at 1 TEOS:0.1 CTABr:0.27 NaOH:1000 H_2_O.

After synthesis, the resulting microparticulated or nanoparticulated powder was recovered by centrifugation, washed with deionized water, and air-dried at room temperature. To prepare the final mesoporous materials, the as-synthesized solids were calcined at 550 °C using an oxidant atmosphere for 5 h in order to remove the template phase.

### 2.3. Mesoporous Silica Particle Loading and Functionalization

Once the starting supports (**M** and **N**) were synthesized, both supports were loaded with rhodamine B (**M-Rh** and **N-Rh**). Amounts of 100 mg of template-free MCM-41 and 39 mg of rhodamine B dye (0.8 mmol Rhodamine B/g MCM-41) were suspended in 25 mL of acetonitrile inside a round-bottom flask in an inert atmosphere. The mixture was then stirred for 24 h at room temperature to achieve maximum loading in the MCM-41 scaffolding pores.

To obtain loaded and functionalized solids (**M-Rh-N3** and **N-Rh-N3**), an excess of N3 (0.43 mL, 0.015 mmol) was added to the mixtures. The final mixtures were stirred for 5.5 h at room temperature. The two loaded and functionalized solids were then isolated by vacuum filtration, washed with 300 mL of water adjusted to pH 2, and dried at room temperature for 24 h.

### 2.4. Characterization of Mesoporous Silica Particles

Mesoporous silica particles were characterized by means of powder X-ray diffraction (PXRD), N_2_ adsorption–desorption isotherms, zeta potential, thermogravimetric analyses, and microscopy.

PXRD was performed on a D8 Advance diffractometer (Bruker, Coventry, UK) using CuKα radiation. N_2_ adsorption–desorption isotherms were recorded with a Micromeritics ASAP 2010 automated sorption analyzer (Micromeritics Instrument Corporation, Norcross, GA, USA). The samples were degassed at 120 °C in vacuum overnight. The specific surface areas were calculated from the adsorption data in the low pressure range using the Brunauer–Emmett–Teller (BET) model. Pore size was determined following the Barrett–Joyner–Halenda (BJH) method. From the XRD and porosimetry studies, the a_0_ cell parameter and wall thickness of the various supports were calculated.

The functionalization degree of different particles was estimated by determining the percentage of organic matter in functionalized particles and confirmed by zeta potential measurements. The percentage of organic matter was determined by thermogravimetric analyses (TGA) on a TGA/SDTA 851e Mettler Toledo balance, using an oxidant atmosphere (air, 80 mL/min) with a heating program consisting of a heating ramp of 10 °C per minute from 393 to 1273 K and an isothermal heating step at this temperature for 30 min. The percentage of lost matter in the 100–750 °C range was used to estimate the functionalization degree since the only organic matter in the particle was due to the anchored amines. To determine the zeta potential (α) of bare and functionalized MSP, a Zetasizer Nano ZS unit (Malvern Instruments, Malvern, UK) was used. Samples were dispersed in distilled water at a concentration of 1 mg/mL. Before each measurement, samples were sonicated for 2 min to preclude aggregation, and the particle dispersions were carefully placed in a folded capillary zeta cell (Malvern Instruments, Malvern, UK). The zeta potential was calculated from the particle mobility values by applying the Smoluchowski model. The average of five recordings is reported as the zeta potential. The measurement was performed at 25 °C. Measurements were performed in triplicate.

For transmission electron microscopy (TEM) analysis, MSPs were dispersed in dichloromethane and sonicated for 2 min to preclude aggregates, and the suspension was deposited onto copper grids coated with carbon film (Aname SL, Madrid, Spain). Imaging of the MSP samples was performed using a JEOL JEM-1010 (JEOL Europe SAS, Croissy, France) operating at an acceleration voltage of 80 kV. Field-emission scanning electron microscopy (FESEM) images were acquired with a Zeiss Ultra 55 (Carl Zeiss NTS GmbH, Oberkochen, Germany) operating at 1.5 mV and a working distance of 5.6 mm. Observations were done in the secondary electron mode.

### 2.5. Preparation and Functionalization of EVOH Films

EVOH-32 and EVOH-44 films were cut into 4-cm^2^ pieces and were sequentially cleaned in iso-propanol, acetone, and deionized water. The EVOH films were sonicated twice with each solvent at 10 min intervals. Clean films were left to dry in Petri dishes inside a desiccator with anhydrous calcium sulfate at room temperature (25 °C) for 12 h.

EVOH-32 and EVOH-44 films were irradiated in open glass Petri dishes for 1, 3, 10, and 15 min under a vacuum ultraviolet (UV) Xe excimer lamp with 6 W at 172 nm (UV-Consulting Peschl España S.L., Valencia, Spain). The films were turned over and exposed to UV light under the same conditions. This method was used to oxidize and create carboxylic acid functional groups on both film surfaces.

In order to select the more suitable EVOH film (EVOH-32 or EVOH-44) for the subsequent attachment of N3-MSP, a quantitative method for determining the number of carboxylic acids created after UV irradiation, the Toluidine Blue O (TBO) dye assay, was carried out. The method used was that described by Hernandez, Tseng, Wong, Stoddart and Zink [22], Uchida, et al. [34], and Kang, et al. [35] with some modifications. In brief, control and UV-treatment films were immersed in a TBO solution (0.5 mM TBO solution in deionized water with the pH adjusted to 10.0 with 0.5 M NaOH) and shaken for 2 h at room temperature (25 °C). Then, the films were rinsed three times with deionized water adjusted to pH 10.0 to remove non-complexed dye. To desorb the complexed dye on the film surfaces, films were submerged in 50 wt% acetic acid solution for 15 min. The absorbance of the acetic acid solutions was measured at 633 nm using a UV–visible light (UV–Vis) spectrophotometer (Agilent 8453 Spectroscopy System), and compared with a standard curve of TBO dye in 50 wt% acetic acid solution.

### 2.6. N3-MSP Deposition

**Figure 1:**
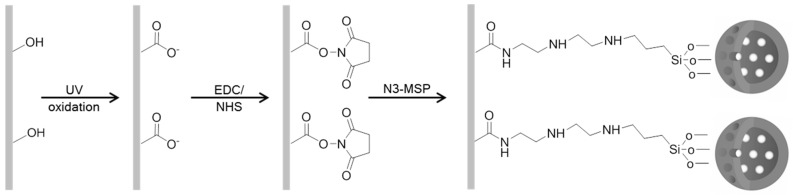
Scheme of the functionalization process: surface modification of ethylene vinyl alcohol (EVOH) and the subsequent carbodiimide-mediated anchoring of *N*-(3-trimethoxysilylpropyl)diethylenetriamine (N3)-functionalized mesoporous silica particles (MSPs).

To attach **M-Rh-N3** and **N-Rh-N3** to the EVOH films via covalent bonding, a previously reported procedure was used [12]. EVOH films were stirred for 30 min in two conjugation buffers containing 5 × 10^−2^ M EDC and 5 × 10^−3^ M NHS at pH 3.0 or 5.0 to select the most adequate bonding conditions. The selected concentrations represent molar excesses of at least 10-fold and 100-fold for EDC and NHS, respectively, compared to the determined mole quantity of surface carboxylic acid groups. Then EVOH films (4 cm^2^/mL) were sonicated for 30 min in buffer (pH 3.0 or pH 5.0) containing **M-Rh-N3** or **N-Rh-N3** at a final concentration of 0.5 mg/mL, and then stirred for 2 h at room temperature (24 ± 1 °C). **EVOH-M-Rh-N3** and **EVOH-N-Rh-N3** films were rinsed with buffer (at pH 3.0 or pH 5.0) to clean unattached particles, before being dried, and stored in dry conditions until use. The whole process is summarized in Figure 1.

### 2.7. Surface Analysis

The efficiency of immobilization of N3-MSP on the EVOH films was studied by means of FE-SEM. FESEM images were acquired with a Zeiss Ultra 55 (Carl Zeiss NTS GmbH, Oberkochen, Germany) and observed in the secondary electron mode. Micrographs of the particles before and after immobilization on the EVOH films were obtained.

### 2.8. Controlled Release from the Films

In a typical experiment, 1 cm^2^ of film was suspended in 4 mL of water adjusted to pH 2 and pH 7.5. At certain times (2 min, 1, 2, 4, 6, 8, and 24 h), aliquots were separated and filtered. Dye released from the pore voids to the aqueous solution was quantified by measuring the emission band of rhodamine B centered at 580 nm (excitation at 554 nm) using a Jasco-FP-8500 spectrofluorometer (Tokyo, Japan).

The rhodamine B release kinetics from pore voids of the porous silica supports were calculated using the Higuchi model, where the amount of guest release, *Q_t_*, per unit of exposed area at time *t* can then be described by the following equation:

$$Q_{t}=k_{H}\;\times \;\sqrt{t},$$

where k_H_ is the release rate constant for the Higuchi model.

## 3. Results and Discussion

### 3.1. MSP Preparation and Characterization

**Figure 2:**
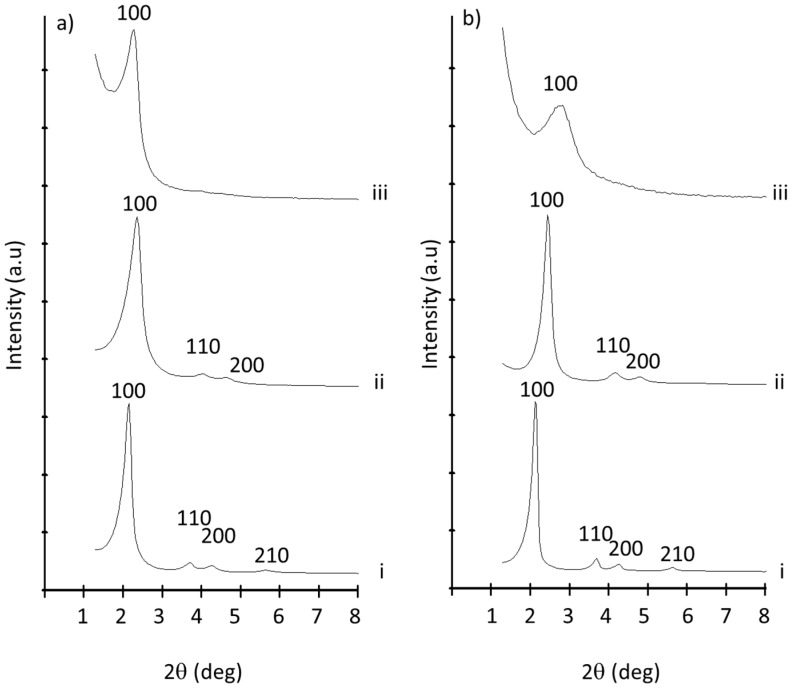
Powder X-ray diffraction of MCM-41 particles as prepared (i), after calcination (ii), and after the loading and functionalization process: (**a**) microparticles; (**b**) nanoparticles.

Microparticulated (**M**) and nanoparticulated (**N**) MSPs as synthesized, MSPs loaded with rhodamine B (**M-Rh and N-Rh**), and MSPs functionalized with *N*-(3-trimethoxysilylpropyl)diethylenetriamine (**N3**) (**M-Rh-N3** for micro and **N-Rh-N3** for nano) were prepared and characterized using standard procedures. Figure 2a shows powder X-ray patterns of the MCM-41 microparticles as synthesized, after calcination, loaded once with Rhodamine B and functionalized with N3 (**M-Rh-N3**).

The PXRD of the microparticulated MSPs as synthesized (Figure 2a, curve i) shows four low-angle reflections typical of a hexagonal array that can be indexed as (100), (110), (200), and (210) Bragg peaks. A significant displacement of the (100) peak in the diffractogram was clearly observed for the calcined microparticles (Figure 2a, curve ii), corresponding to a cell contraction of ca. 4 Å. This displacement and the broadening of the (110) and (200) peaks are most likely related to further condensation of silanol groups during the calcination step. Figure 2a, curve iii corresponds to the **M-Rh-N3** PXRD pattern. In this case, a slight intensity decrease and a further broadening of the (110) and (200) reflections were observed, probably due to a loss of contrast owing to the filling of the pore voids with the dye and the functionalization with amines. Nevertheless, the value and intensity of the (100) peak in this pattern clearly showed that both the loading process with the dye and the subsequent functionalization with amines did not damage the mesoporous scaffolding. The same diffractogram features were obtained for the solid materials prepared with MCM-41 nanoparticles (Figure 2b). Since both particles belong to the MCM-41 family, the similarity between diffractograms of micro- and nanoparticles was expected [31].

**Figure 3:**
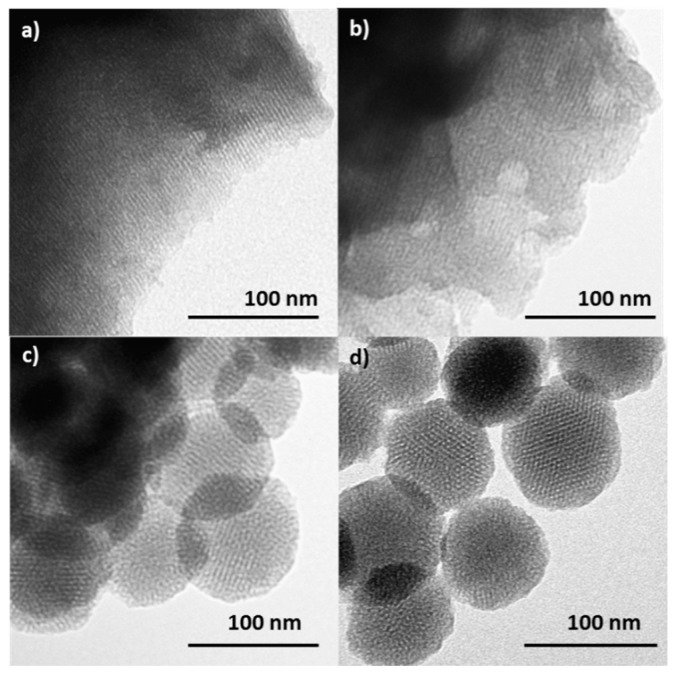
TEM images of calcined (**a**) microparticles (**M**), and (**c**) nanoparticles (**N**), showing the typical hexagonal porosity of the MCM-41 matrix. TEM images of solid MSP micro- and nanoparticles loaded with rhodamine B and functionalized with N3: (**b**) **M-Rh-N3**, and (**d**) **N-Rh-N3**.

The preservation of the mesoporous structure in the final loaded and functionalized solids **M-Rh-N3** and **N-Rh-N3** was also confirmed by means of transmission electron microscopy (TEM). Figure 3 shows the different morphologies of the two types of particles. While MCM-41 microparticles (Figure 3a) are irregular particles with diameters ranging between 0.8 and 1.2 mm, MCM-41 nanoparticles (Figure 3c) show a spherical shape with diameters of ca. 100 nm. No significant differences were observed in particle size before and after functionalization. The images show the typical channels of the MCM-41 matrix both as alternate black and white stripes and as a pseudo-hexagonal array of pore voids in both types of particles. These channels are seen not only in the calcined material but also in the loaded and functionalized supports (Figure 3b,d), confirming that the initial morphology of the mesoporous matrix was maintained after the loading and functionalization process.

Textural properties of the various supports calculated from the nitrogen adsorption–desorption isotherms are summarized in Table 1. As observed, both types of particles (micro and nano) presented similar textural properties (total area of ca. 1000 m^2^·g^−1^, pore volume of ca. 0.9 c^3^·g^−1^, and pore size of ca. 2.5 nm). These features were reported to be sufficient for encapsulation of molecules of special interest in food technology (i.e., antimicrobial agents, drugs, flavors, vitamins, antioxidants, enzymes, and other functional compounds) in MSPs [36]. Table 1 also shows that, after the loading and functionalization process, a decrease in the N_2_ volume adsorbed was produced. This reduction is indicative of mesoporous systems with partially filled mesopores.

The content of organic matter in the final hybrid solids **M-Rh-N3** and **N-Rh-N3** was determined by thermogravimetric analysis. Contents (α) of rhodamine B and the amine derivative are shown in Table 1. The organic matter contents in both materials is similar to those reported by other authors for similar systems based on MSPs loaded with rhodamine B and functionalized with amines [37]. Table 1 also includes the zeta potential values of **M-Rh-N3** and **N-Rh-N3** suspended in distilled water. Bare micro- and nanoparticles showed negative zeta potential values of ca. −35 mV. These negative values are typical of bare mesoporous silica particles, which contain SiO– groups in their surfaces. After functionalization with amines, a neutralization of the silica by ammonium groups was produced, and zeta potential values changed from negative to positive values (ca. +40 mV). This inversion of the surface charge after organic functionalization was reported in literature for similar systems [38,39] and confirms the efficiency of the functionalization process.

### 3.2. EVOH Film Surface Analysis

Two EVOH copolymers with a different molar percentage of ethylene monomer (32%—EVOH 32, and 44%—EVOH 44) were selected as films for functionalization. The lower the ethylene content, the higher the hydrophilicity, the interchain interactions, the rigidity, and the gas barrier. The films were supplied as the central layer of a three-layer polypropylene (PP)/EVOH/PP coextruded film without tie layers, so the PP protector layers could be easily peeled off. Monolayer EVOH films were exposed to UV irradiation to oxidize the film surface. The presence of a significant amount of –OH substituents permits the generation of carboxylic substituents, which are required for **M-Rh-N3** and **N-Rh-N3** particle attachment via EDC/NHS.

**Figure 4:**
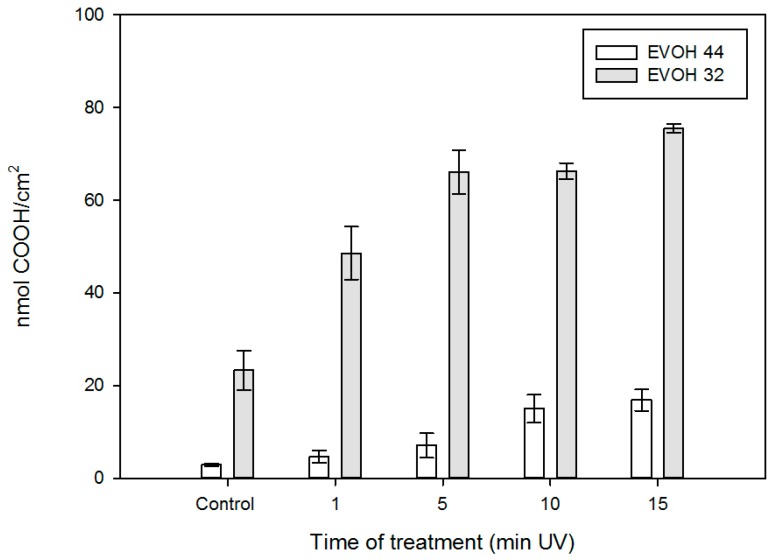
Surface density of COOH groups on EVOH 32 and EVOH 44 films calculated by the Toluidine Blue O (TBO) assay as a function of the ultraviolet (UV) treatment time.

The TBO assay was used to quantify the amount of carboxyl groups created after the irradiation on the surface of the EVOH 32 and EVOH 44 films (Figure 4) [40]. The non-zero initial relevant value for EVOH 32 is due to the presence of some carbonyl groups in the copolymer, as this family of materials is obtained via hydrolysis of statistical poly(ethylene-*co*-vinyl acetate) polymers.

As expected, the density of carboxylic groups increased significantly for both films with the length of UV treatment. For instance, the number of nmol of COOH/cm^2^ for EVOH 32 films ranged from 23.31 nmol/cm^2^ after 1 min to 75.49 nmol/cm^2^ after 15 min of UV treatment. A similar trend was found for the EVOH 44 samples, although, in this case, the density of the generated carboxyl groups was significantly lower than that found for EVOH 32.

### 3.3. MSP Immobilization

**Figure 5:**
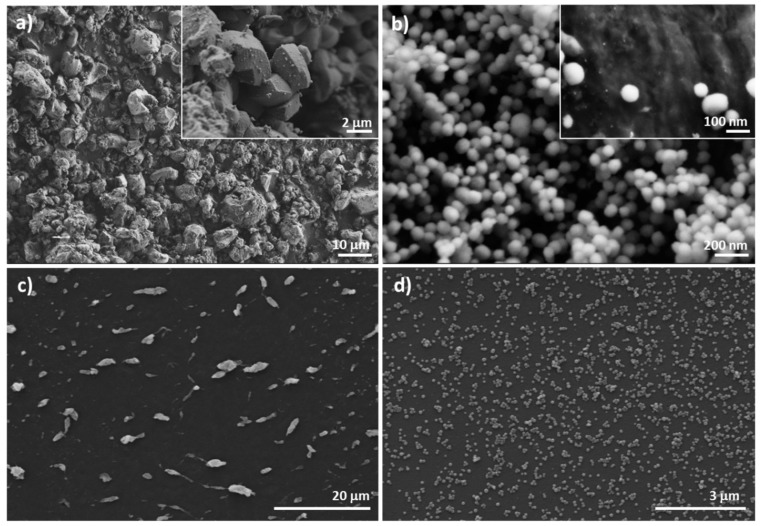
Representative field-emission SEM (FESEM) images of (**a**) **M-Rh-N3** and (**b**) **N-Rh-N3** particles, and (**c**) **M-Rh-N3** and (**d**) **N-Rh-N3** particles covalently anchored to EVOH 32 film after being UV-treated for 15 min.

**Figure 6:**
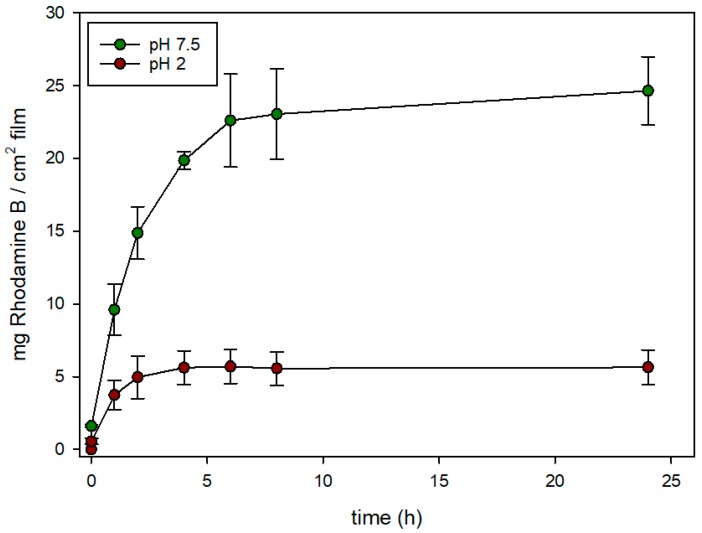
Evolution of rhodamine B release from **EVOH 32-N-Rh-N3** prepared at pH 3 in aqueous media at acidic pH (pH = 2) and at neutral pH (pH = 7.5).

**Figure 7:**
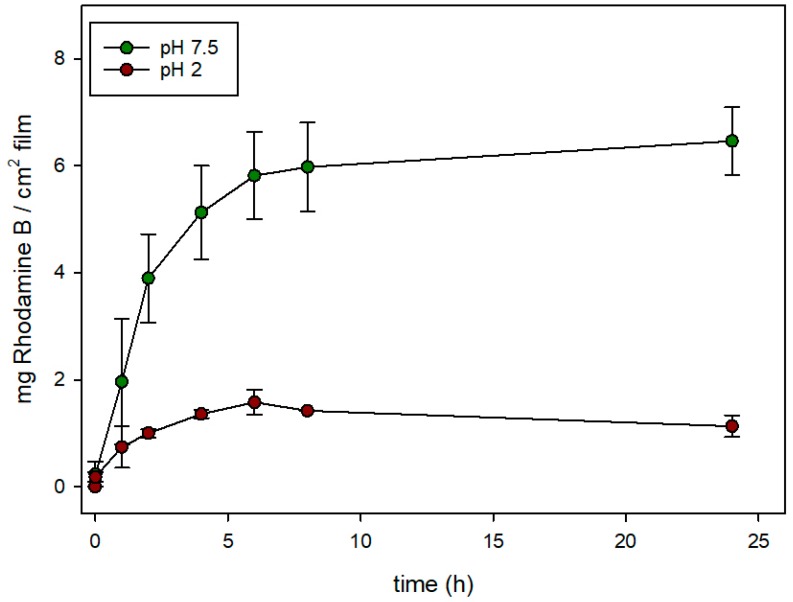
Evolution of rhodamine B release from **EVOH 32-M-Rh-N3** prepared at pH 3 in aqueous media at acidic pH (pH = 2) and at neutral pH (pH = 7.5).

After confirming the activation of the EVOH films, the amine-functionalized mesoporous silica particles were covalently linked to the films through amide bonds in the presence of carbodiimide. The immobilization efficiency of **M-Rh-N3** and **N-Rh-N3** on the surface of the EVOH films was studied by means of FESEM. Micrographs of the gated MSPs before and after immobilization on the films are shown in Figure 5. Four films were developed, **EVOH 32-N-Rh-N3** in which the nanoparticles were anchored at pH = 5 (**FN5**) and at pH = 3 (**FN3**) (Figure 6), and **EVOH 32-M-Rh-N3** anchored at pH = 5 (**FM5**) and at pH = 3 (**FM3**) (Figure 7). As previously commented (Figure 2), the micro- and nanoparticles exhibited differences with regard to size and shape (Figure 5a,b). The micro MSPs were irregular in shape and size, whereas the nano MSPs appeared as equal spheres of ca. 100 nm. These particle differences conditioned the way in which the MSPs anchored on the films. Figure 5c,d shows the distribution of micro- and nanoparticles on the EVOH 32 film surface UV-treated for 15 min after reaction of the EVOH 32 film with an equal mass of **M-Rh-N3** or **N-Rh-N3**. A much better dispersion was observed for the nanoparticles (Figure 5d) when compared with the microparticles (Figure 5c). Moreover, the number of particles attached to the surface of the film was estimated as 0.05 ± 0.01 particles/µ^2^ for **M-Rh-N3** and 67 ± 5 particles/µ^2^ for **N-Rh-N3.** Similar results, although with a much lesser immobilization of particles, were obtained for the EVOH 44 films (data not shown). Therefore, the EVOH 44 films were discarded.

### 3.4. Controlled Release Behavior

Finally, and with the objective of confirming the efficiency of the gated materials for releasing rhodamine B, controlled delivery studies from the functionalized films according to the pH in water (used as an aqueous food simulant) were carried out.

Figure 6 and Figure 7 show the release rates of rhodamine B from **EVOH 32-N-Rh-N3 and EVOH 32-M-Rh-N3** films functionalized at pH 3 (expressed as mg of rhodamine B released from 1 cm^2^ of film) when immersed in two aqueous food simulants, at acidic (pH = 2) and neutral pH (pH = 7.5). In **EVOH 32-N-Rh-N3** films (Figure 6), the maximum release of rhodamine B was observed at neutral pH. In these conditions, a controlled, sustained release was achieved during 8 h reaching a maximum delivery content of ca. 24.5 mg of rhodamine B per cm^2^ of film Conversely, when this sample was exposed to an acidic medium, the release was significantly reduced (maximum delivery of ca. 5.5 mg of rhodamine B per cm of film), reaching a flat baseline during the first hours of the experiment. This different and remarkable behavior at pH 7.5, when compared to that at pH 2, was due to the effect of pH on the conformation of the polyamines. This gating-mechanism was widely described in recent years [33]. At pH 2, polyamines are protonated into polyammonium groups that favor Coulombic repulsions between close chains. Tethered polyammonium moieties tend to adopt a rigid-like conformation that pushes them away toward pore openings, blocking the pores and completely or partially inhibiting release of the sorbed substance. In contrast, a progressive delivery of the colorant was observed at pH 7.5. In this condition (neutral pH), polyamines are less protonated and their repulsions are weaker, favoring a pore unblockage that allows the release of the encapsulated fluorophore.

Similar behavior can be seen in Figure 7 for **EVOH 32-M-Rh-N3**. A sustained release of the cargo was observed after adding water adjusted to pH 7.5 to the films, while release was hindered after the addition of water adjusted to pH 2. Again, at neutral pH, polyamines are not protonated and their interactions are weaker, favoring pore unlock. However, despite the fact that MCM-41 nano- and microparticles were loaded with similar amounts of rhodamine B (see Table 1), the amount of rhodamine delivered from **EVOH 32-M-Rh-N3** was ca. four-fold lower than that achieved from **EVOH 32-N-Rh-N3** films. This difference might be explained by the different density of particles anchored to the film (see Figure 4).

Finally, the results presented in this work also confirm that amines do not lose their gating properties after immobilization on EVOH films, opening a new field for controlled release in packaging applications.

**Figure 8:**
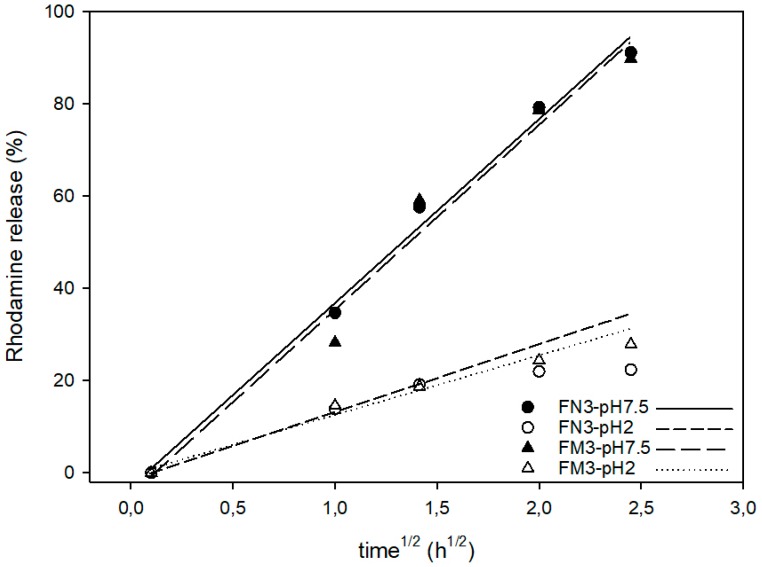
Higuchi representation of rhodamine B release from **EVOH 32-M-Rh-N3** and **EVOH 32-N-Rh-N3** prepared at pH 3 in aqueous media at acidic pH (pH = 2) and at neutral pH (pH = 7.5). Symbols correspond to experimental data, and the lines are the fitting lines of the Higuchi equation.

To compare the rhodamine B release kinetics from pore voids of both types of silica supports (nano and micro) the experimental release data were fitted to the Higuchi model, and the Higuchi release rate constant (k_H_) was calculated. The good fit of the delivery curves to the Higuchi model, as Figure 8 shows, suggests that the delivery of rhodamine B from the pores of various solids is basically a diffusive process [31]. Moreover, for both types of particles, the k_H_ constant at pH 7.5 was the same (k_H_ = 40). There were also no significant differences in Higuchi rate constants at acidic conditions: k_H_ was 15 for **EVOH 32-N-Rh-N3,** and 13 for **EVOH 32-M-Rh-N3.** These data confirm that delivery at pH 7.5 is not only more efficient than at pH 2, but also faster. Moreover, the small differences between the two types of particles demonstrate that release kinetics is influenced by the porous system (equal in all MCM-41 particles) instead of by the particle morphology. Accordingly, differences observed in the comparison of Figure 6 with regard to the amount of dye released by the encapsulation systems (20%–30% greater in **EVOH 32-N-Rh-N3** films) are due to the higher concentration of particles achieved during the N3-MSP deposition step (see Figure 4).

## 4. Conclusions

This work is a proof of concept for the design of active packaging materials with a pH triggering mechanism. Mesoporous silica micro- and nanoparticles (MCM-41) were manufactured, calcined, and used to load and release an agent in a controlled manner when exposed to suitable pH conditions. The particles were loaded with rhodamine B (selected as a dye whose release is easy to monitor) and functionalized with *N*-(3-trimethoxysilylpropyl)diethylenetriamine (polyamines). This functionalization creates chemical gates whose key is based on pH.

In parallel, poly(ethylene-*co*-vinyl alcohol) (EVOH) films with two monomer compositions, EVOH 32 and EVOH 44, were successfully oxidized by UV irradiation. The treatment generated –COOH substituents in the polymer chains, which increased with an increase in irradiation time and a decrease in copolymer ethylene content. Oxidized EVOH was used to anchor the loaded silica particles through the use of EDC/NHS linkers. Linkage was carried out successfully at pH 3 and pH 5, especially for nanoparticles, which were distributed homogeneously throughout the film surface, especially in the case of EVOH 32 films.

Finally, the ability to keep and release the agent was analyzed. The final load of the dye was greater in the films exposed to anchorage treatments at pH 3, as, at pH 5, a partial release of rhodamine B was evidenced during the process. The films with the anchored particles were exposed to two liquid media, simulating acidic food and neutral food. The films released the agent quickly and completely at neutral pH, but kept the dye locked at acidic pH.

Hence, this work demonstrates the feasibility of covalently anchoring smart delivery systems able to deliver a functional molecule after applying a triggering stimulus to EVOH films. This successful mechanism will allow the design of new active packaging systems loaded with antioxidant, antimicrobial, or aromatic agents able to release their cargo only under certain conditions, such as the generation of biogenic amines by bacteria in fresh food products.

## Figures and Tables

**Table 1:** Characterization of the mesoporous silica particles (MSPs) before and after functionalization: Brunauer–Emmett–Teller (BET) specific surface area, pore volume, and pore size calculated from the N_2_ adsorption–desorption isotherms, content of rhodamine B and amines (α_Rh_ and α_N3_, mg/g_solid_), and zeta potential (Z-potential, mV). M—microparticles; N—nanoparticles; Rh—loaded with rhodamine B; N3—functionalized with *N*-(3-trimethoxysilylpropyl)diethylenetriamine.

Sample	BET Area (m^2^/g)	Pore Volume (c^3^/g)	Pore Size (nm)	α_Rh_ (mg/g_solid_)	α_N3_ (mg/g_solid_)	Z-Potential (mV)
**M**	1072	0.91	2.62	-	-	−38
**M-Rh-N3**	243	-	-	15.3	81	41
**N**	986	0.84	2.51	-	-	−36
**N-Rh-N3**	143	-	-	17.2	142	43
